# Effect of Renin-Angiotensin-Aldosterone System Inhibitors in Patients with COVID-19: a Systematic Review and Meta-analysis of 28,872 Patients

**DOI:** 10.1007/s11883-020-00880-6

**Published:** 2020-08-24

**Authors:** Ranu Baral, Madeline White, Vassilios S Vassiliou

**Affiliations:** 1 Norfolk and Norwich University Hospital, Norwich, UK; 2 Norwich Medical School, University of East Anglia, Norwich Research Park, Norwich, NR4 7UQ UK

**Keywords:** Renin-angiotensin-aldosterone system, Hypertension, Coronavirus, COVID

## Abstract

**Purpose of Review:**

The role of renin-angiotensin-aldosterone system (RAAS) inhibitors, notably angiotensin-converting enzyme inhibitors (ACEi) or angiotensin receptor blockers (ARBs), in the COVID-19 pandemic has not been fully evaluated. With an increasing number of COVID-19 cases worldwide, it is imperative to better understand the impact of RAAS inhibitors in hypertensive COVID patients. PubMed, Embase and the pre-print database Medrxiv were searched, and studies with data on patients on ACEi/ARB with COVID-19 were included. Random effects models were used to estimate the pooled mean difference with 95% confidence interval using Open Meta[Analyst] software.

**Recent Findings:**

A total of 28,872 patients were included in this meta-analysis. The use of any RAAS inhibition for any conditions showed a trend to lower risk of death/critical events (OR 0.671, CI 0.435 to 1.034, *p* = 0.071). Within the hypertensive cohort, however, there was a significant lower association with deaths (OR 0.664, CI 0.458 to 0.964, *p* = 0.031) or the combination of death/critical outcomes (OR 0.670, CI 0.495 to 0.908, *p* = 0.010). There was no significant association of critical/death outcomes within ACEi vs non-ACEi (OR 1.008, CI 0.822 to 1.235, *p* = 0.941) and ARB vs non-ARB (OR 0.946, CI 0.735 to 1.218, *p* = 0.668).

**Summary:**

This is the largest meta-analysis including critical events and mortality data on patients prescribed ACEi/ARB and found evidence of beneficial effects of chronic ACEi/ARB use especially in hypertensive cohort with COVID-19. As such, we would strongly encourage patients to continue with RAAS inhibitor pharmacotherapy during the COVID-19 pandemic.

**Electronic supplementary material:**

The online version of this article (10.1007/s11883-020-00880-6) contains supplementary material, which is available to authorized users.

## Introduction

Coronavirus disease 2019 (COVID-19), emerging from Wuhan, China, in December 2019 has quickly evolved into a global pandemic. It is caused by severe acute respiratory syndrome coronavirus 2 (SARS-CoV-2) [1] and affects all the organs of the body and especially the lungs. As of 20th May 2020, WHO reported 4,789,205 cases of COVID-19 worldwide and 318,789 deaths [2].

In such an unprecedented pandemic, the role of renin-angiotensin-aldosterone system (RAAS) inhibitors, notably angiotensin-converting enzyme inhibitors (ACEi) or angiotensin receptor blockers (ARBs), in COVID-19 has been questioned. The particular concern emerged given the significant role of ACE2 as a receptor for SARS-COV-2, which enables entry into host cells [3]. Considering the substantial expression of ACE2 receptors in the respiratory and cardiovascular system, it is not a surprise that SARS-COV-2 causes not only respiratory, but also extensive cardiac injury [4]. The chronic use of RAAS inhibitors has been speculated to increase the levels of ACE2 and potentially exaggerate the severity of COVID-19 with early reports supporting this [3].

RAAS inhibitors, although primarily used for hypertension, are indicated in other cardiovascular patients including those with prior myocardial infarction, heart failure, cerebrovascular disease or chronic kidney disease [5]. The patients with cardiovascular diseases are at particular risk of COVID-19 infections [6, 7]. Hence, with an increasing number of COVID-19 cases worldwide and the likelihood of a ‘second wave’ of infection, it is imperative to better understand the impact RAAS inhibitor use in COVID-19 patients. We, thus, conducted an up-to-date systematic review and meta-analysis of RAAS blockers in patients with COVID-19.

## Methods

### Search Strategy

The systematic review was conducted and reported in accordance with the Preferred Reporting Items for Systematic Reviews and Meta-Analyses (PRISMA) guidelines. PubMed and Embase and pre-print database Medrxiv were searched from inception to 17 May 2020 using key terms such as ‘Angiotensin-Converting Enzyme inhibitors’, ‘Angiotensin Receptor Blockers’, ‘coronavirus disease 2019’, and ‘SARS-COV-2’. The full search strategy is included in (Supplementary Figure 1). Studies published in languages other than English were excluded. A snowballing method was used to the references of retrieved papers to expand the search.

### Inclusion and Exclusion Criteria

All studies identified in our search were screened using the titles and the abstracts. Duplicate studies and multiple reports from same studies were removed. Any article identified as having a potential of fulfilling our inclusion criteria underwent full-text evaluation. Any study design, except for narrative reviews or opinion-based publications, with ACEi/ARB data on adult (≥ 18 years) patients with COVID-19 was included, and relevant information such as type of study, characteristics of patients, mortality and data relating to clinical severity of COVID-19 infection was extracted.

The proportion of COVID-19 patients on ACEi/ARB and their mortality and clinical severity data was compared to non-ACEi/ARB patients. We only included deaths and ‘critical’ events in our analysis defined as ITU admission and invasive and non-invasive ventilation. Data for severe outcomes [8] including high-flow oxygen use but in a non-ITU [1] setting were excluded. Where studies included more than one outcome of ‘critical’ events, e.g. ITU admission and ECMO use, we only considered the lowest qualifying criterion to avoid double-counting of patients.

### Statistical Analysis

The data was analysed using random effects in Open Meta[Analyst] software version 10.12 (developed by the Centre for Evidence Synthesis, Brown University, School of Public Health, RI, USA) [9]. Statistical heterogeneity was evaluated by calculating *I*^2^ statistics. The statistical significance was defined as *p* < 0.05.

### Publication Bias

Funnel plots were used to assess publication bias using Review Manager (RevMan) software (Version 5.3. Copenhagen: The Nordic Cochrane Centre, The Cochrane Collaboration, 2014).

### Study Quality

The Newcastle-Ottawa Scale (NOS), a nine-point scale to assess the quality of cohort and case control/case-series, was used to evaluate the included studies.

## Results

Our search yielded 1031 studies from the database (PubMed and Embase) searches (Supplementary Figure 2). After de-duplication, we rejected 666 trials after title-abstract screening. A total of forty trials underwent full-text evaluation. Trials including clinically suspected COVID-19 patients but without a positive test [10] or no original data were excluded. A total of twenty studies were thus included in meta-analysis (Table 1). Following submission of our article, one study [6] was retracted [11], and therefore we excluded this from our analysis.

**Table 1:** Baseline characteristics of included studies

Authors	Source	Description of study	Outcomes	ACEi/ARB	Total patients	Characteristics of total patients	Subgroup	ACEi/ARB in subgroup	Characteristics of subgroup patients
Abajo	PubMed	Case-population study in Madrid, Spain	Prevalence of ACEi/ARB	ACEi: 240 ARB: 244	1139	Female: 39.0% HTN: 54.2% DM: 27.2% HF: 7.0% Stroke/TIA: 6.4% Cardiovascular disease: 27.4%	-	-	-
Andrea	PubMed	Retrospective, observational single-centre case series in Milan, Italy	Survival data. Median follow-up 28 days	69	191	Female: 31.4% Age (mean): 63.4 ± 14.9 CHD:14.7% DM: 14.7% HTN: 50.2% HF: 4.7%	HTN: 96	68	CHD: 28.1% DM: 22.9% HTN: 100% HF: 8.3%
Bean	Medxiv	Multi-centre cohort study of COVID-19 inpatients in London, UK	Survival and critical care admission. Follow-up 21 days	399	1200	Female: 42.8% Age (mean): 68.0 ± 17.07 Stroke/TIA: 19.6 IHD: 13.3% DM: 34.8% HTN: 53.8% HF: 8.9%	-	-	-
Chen	PubMed	Retrospective study of COVID-19 inpatients in central hospital of Wuhan, China	Length of hospital stay, clinical outcome: discharge or death in hospital.	NR	341	Female: 46.3% Age (median): 58 (42.0–62.0) DM: 14.4% HTN: 36.7% Cardiovascular: 14.7%	HTN+ DM: 71	32	Age (median): 67.0 (61.0–76.0) DM: 100% HTN: 100%
Chocdik	PubMed	Observational study of 1 COVID-19 inpatients identified using Maccabi Health Services database Israel	Prevalence of ACEi/ARB	ACEi: 55 ARB: 76	1317	Female: 40.2% Age (mean): 40.6 ± 19.1 DM: 8.7% HTN: 14.0% HF: 0.2%	-	-	-
Dauchet	Medxiv	Mono-centric study of in-patients and outpatients of Lille, France	Critical care admission	ACEi: 31 ARB: 31	187	NR	-	-	-
Feng	PubMed	Multi-centre retrospective, observational study of COVID-19 inpatients in China Wuhan, Shanghai and Anhui	Survival, severity of disease based on CCDC*	NR	476	Female: 46.1% Age (median): 53(40–64) CVD: 3.6% DM: 10.3% HTN: 23.7% Cardiovascular: 7.9%	HTN: 113	33	NR
Guo	PubMed	Retrospective single-centre case series of COVID-19 inpatients in Wuhan City, China	Prevalence of ACEi/ARB	19	187	Female: 51.3% Age (mean): 58.5 ± 14.7 CHD: 11.2% DM: 15.0% HTN: 32.6%	-	-	-
Huang	PubMed	Observational, single-centre study of COVID-19 inpatients with HTN in Wuhan, China	Non-invasive (+ high flow oxygen), invasive ventilation, death, ECMO, severity based on CCDC*	-	-	-	HTN: 50	20	Female: 46.0% HTN: 100%
Ip	Medxiv	Retrospective, multi-centre study with convenience sampling of COVID-19 inpatients in USA	Survival data	NR	3017	NR	HTN: 1129	460	NR
Li	PubMed	Single-centre, observational, case series of COVID-19 inpatients with HTN in Wuhan, China	Mortality ARDS Length of hospital stay Severity based on CCDC*	NR	1178	Female: 53.7% CVD: 8.1% CHD: 8.7% DM: 17.2% HTN: 30.1% HF: 1.8%	HTN: 362	115	Female: 47.8% Age (median): 66 (59–73) CVD: 18.8% CHD: 17.1% DM: 35.2% HTN: 100% HF: 2.8%
Mancia	PubMed	Population-based, case-control study in the Lombardy, Italy	Critical/fatal infection who had assisted ventilation or died	ACEi: 1502 ARB: 1394	6272	Female: 36.7% Age (mean): 68 ± 13 CHD:7.5% HF: 5.1% Cardiovascular: 30.1%	-	-	-
Mehra	PubMed	Multi-centre observational study in 169 hospitals in Asia, Europe and North America	Survival data	ACEi: 770 ARB: 556	8910	Female: 40.0% Age (mean): 49 ± 16 CHD:11.3% HTN: 26.3% HF: 2.1% Cardiovascular: 30.1%	-	-	-
Mehta	PubMed	Retrospective, cohort study of all patients tested for COVID-19 at the Cleveland Clinic Health System in Ohio and Florida	Intensive care admission, ventilation, hospital admission	ACEi: 116 ARB: 98	1735	NR	-	-	-
Meng	PubMed	Retrospective, single-centre review of COVID-19 inpatients admitted to the Shenzhen Third People’s Hospital in China	Mortality Severity based on CCDC*	NR	417	NR	HTN 42	17	Female: 42.9% Age (median): 64.5 (55.8–69) CHD: 19.0% DM: 14.2% HTN: 100%
Reynolds	PubMed	Observational study of people who were tested for COVID-19 using New York University (NYU) Langone Health record	Likelihood of positive test and severe outcomes	NR	5894	NR	HTN 2573	NR	NR
Richardson	PubMed	Multi-centre case series of patients with COVID-19 inpatients in New York, USA	Death	456	2411	NR	HTN 1366	ACEi: 189 ARB: 267	Female: 39.7% Age (median): 63(52–75) CHD:11.1% DM: 33.8% HTN: 56.6% HF: 6.9%
Yan	Medxiv	Multi-centre, case-control study of COVID-19 inpatients Zhejiang province, China	Clinical outcomes; severity based on CCDC*	ACEi: 5 ARB: 53	610	Female: 48.9% Age (mean): 48.8 ± 14.2 Cardio or cerebro disease: 2.62% DM: 9.8% HTN: 22.5%	-	-	-
Yang	PubMed	Retrospective, single-centre, case-control study of COVID-19 inpatients with HTN in Wuhan, China	Death, severity based on CCDC and length of hospital stay	NR	462	NR	HTN 126	43	Female: 50.8% Age (median): 66(61–73) DM: 30.2% HTN: 100%
Zhang	PubMed	Observational, retrospective, multi-centre cohort study in Hubei, China, of HTN patients with COVID-19	Death, clinical outcomes: ARDS, DIC, AKI, acute heart injury septic shock NIV, IV, ECMO Follow-up 28 days	NR	3430	NR	HTN: 1128	188	Female: 46.5% Age (median): 64- CVD: 3.6% CHD: 11.6% DM: 21.2% HTN: 100%

*CCDC: Severity assessed according to Chinese Center for Disease Control and Prevention reports

*ACEi*, angiotensin-converting enzyme inhibitor; *ARB*, angiotensin receptor blocker; *HTN*, hypertension; *DM*, diabetes mellitus; *CVD*, cerebrovascular disease; *ARDS*, acute respiratory distress syndrome; *DIC*, disseminated intravascular coagulopathy; *AKI*, acute kidney injury; *NIV*, non-invasive ventilation; *IV*, invasive ventilation; *HF*, heart failure; *COVID-19*, coronavirus disease; *TIA*, transient ischaemic attack; *ECMO*, extra corporeal membrane oxygenation

Most studies were retrospective, observational [3, 12–15], multi-centre studies mainly conducted in China [3, 12, 16–18]. There were no randomised controlled studies. Many studies included mortality data for a subgroup, commonly hypertensive patients in their analysis. One study used cardiovascular patients and the other studies included hypertensive patients with diabetes. All included trials scored six or higher than 6 (moderate to high) in the Newcastle-Ottawa Scale (NOS) (Supplementary Table 1).

A total of 27.9% (8041/28872) of the patients with COVID-19 were on ACEi/ARBs (Table 1). Among hypertensive COVID-19 patients, 32.3% (3140/9706) were on ACEi/ARB.

**Fig. 1:**
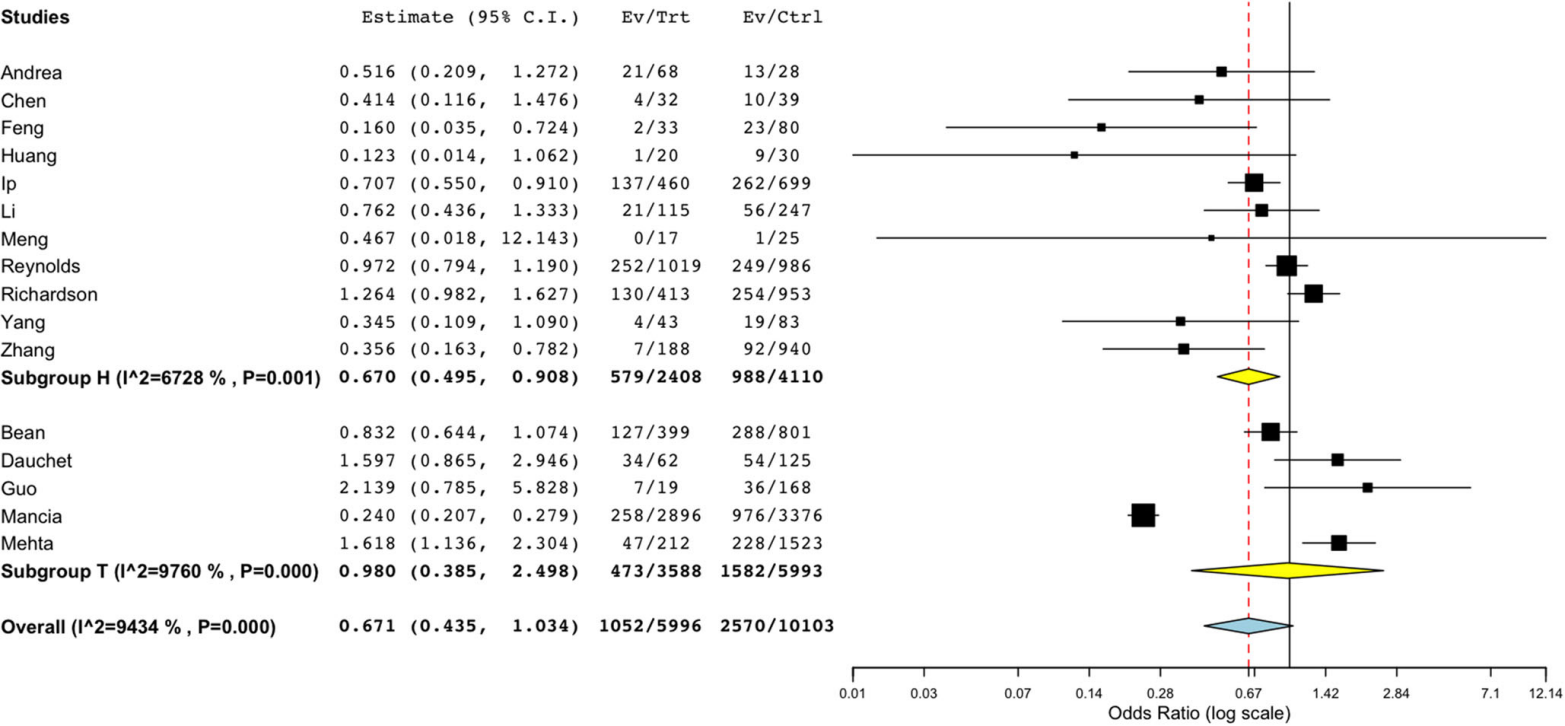
Subgroup analysis of death/critical events in ACEi/ARB vs non-ACEi/ARB. Subgroup analysis of death/critical events (OR 0.671, CI 0.435 to 1.034, *p* = 0.071) in sixteen studies with 5996 patients on ACEi/ARB vs 10,103 non-ACEi/ARB patients. Total effect for subgroup H with 11 studies (OR 0.670, CI 0.495 to 0.908, *p* = 0.010). Subgroups *H* and *T* refer to reference population; *H* is hypertension, *T* for sample population with mixed comorbidities. *I^2* refers to *I*^2^ as a measure of heterogeneity

Most studies categorised clinical outcomes for patients as ‘critical’ or ‘severe’ [3, 12, 16, 17] assessed using Chinese Center for Disease Control and Prevention report [19]. The patients with at least ‘critical’ clinical outcome or need for intensive care or who died were included in this analysis. In a pooled analysis of 16,099 patients in sixteen studies, there was a trend towards a reduction in the odds of death/critical outcomes in those on ACEi/ARB as compared to those not on ACEi/ARB (pooled OR 0.671, CI 0.435 to 1.034, *p* = 0.071) as shown in Fig. 1. Importantly among hypertensive patients in eleven studies (subgroup H), there was a significantly lower risk of death/critical outcomes (OR 0.670, CI 0.495 to 0.908, *p* = 0.010) (Fig. 1) confirming the safe chronic use of ACEi/ARB and an association with better outcomes. Sensitivity analysis of death/critical events for both groups together (hypertensive and non-hypertensive patients) rendered the overall results significant when each of four studies [7, 14, 20, 21•] was removed individually (Supplementary Figures 4–7). However, no significant changes were seen in the overall population when any of the other studies was excluded. Meta-regression, in addition to subgroup analyses, was done to estimate the effect of hypertension as a covariate which was not significant (*p* = 0.205).

**Fig. 2:**
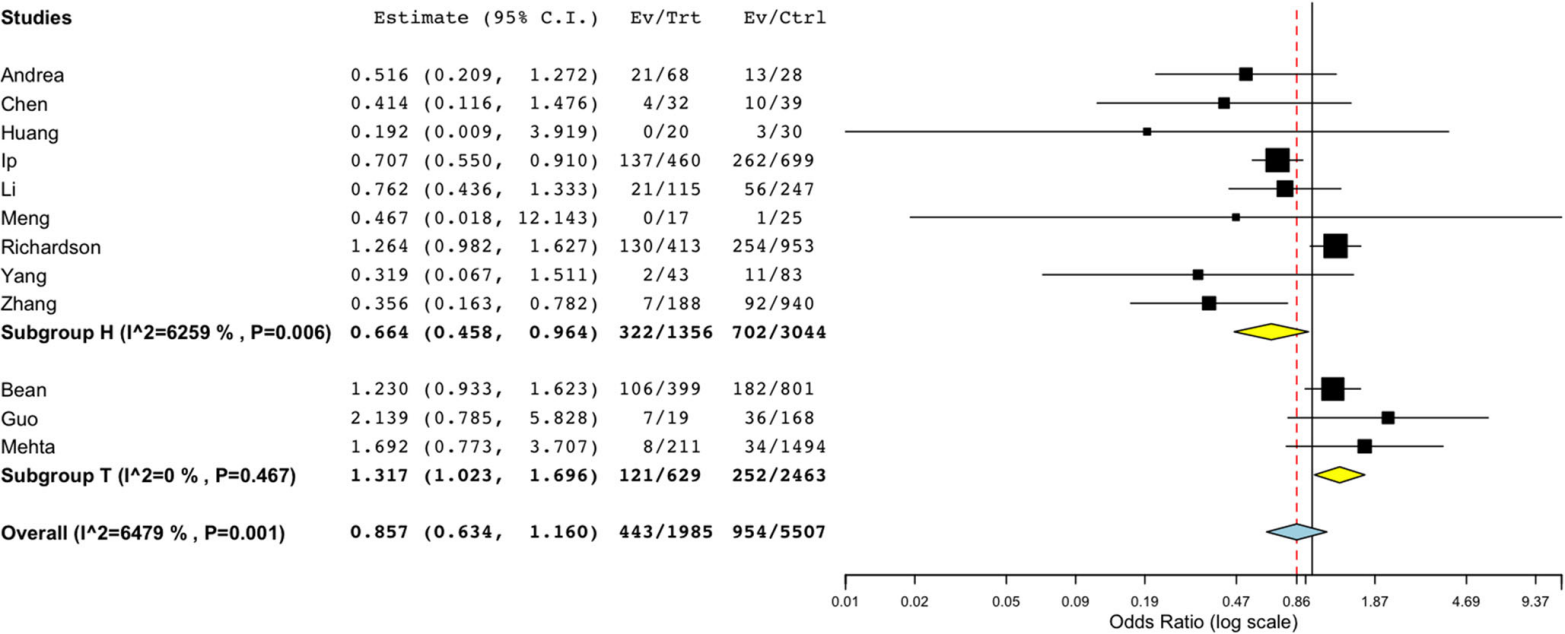
Subgroup analysis of death in ACEi/ARB vs non-ACEi/ARB. Subgroup analysis of death in twelve studies (OR 0.857, CI 0.634 to 1.160, *p* = 0.318) in ACEi/ARB vs non-ACEi/ARB. Subgroup H with nine studies (OR 0.664, CI 0.458 to 0.964, *p* = 0.031).Subgroups *H* and *T* refer to reference population; *H* is hypertension; *T* for sample population with mixed comorbidities. *I^2* refers to *I*^2^ as a measure of heterogeneity

A total of twelve studies reported death in patients taking ACEi/ARB vs non-ACEi/ARB. The meta-analysis demonstrated no increased risk of death in patients taking ACEi/ARB (pooled OR 0.857, CI 0.634 to 1.160, *p* = 0.318) as shown in Fig. 2. Among the hypertensive cohort (subgroup H), there was a statistically significant reduction in the odds of death/critical events in patients taking ACEi/ARB (OR 0.664, CI 0.458 to 0.964, *p* = 0.031).

**Fig. 3:**
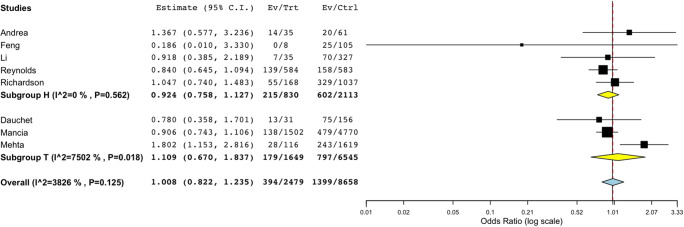
Subgroup analysis of death/critical events in ACEi vs non-ACEi. Subgroup analysis of death/critical events in eight studies (OR 1.008, CI 0.822 to 1.235, *p* = 0.941) in ACEi vs non-ACEi. Subgroups *H* and *T* refer to reference population; *H* is hypertension, *T* for sample population with mixed comorbidities. *I^2* refers to *I*^2^ as a measure of heterogeneity

**Fig. 4:**
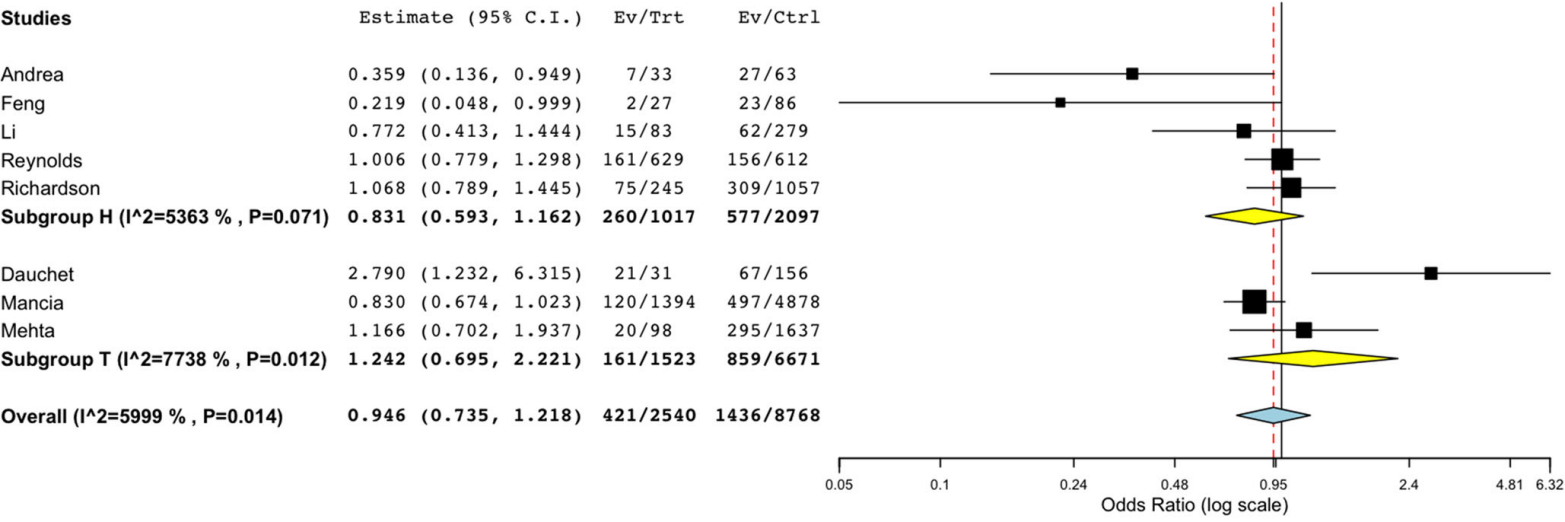
Subgroup analysis of death/critical events in ARB vs non-ARB. Subgroup analysis of death/critical events in eight studies (OR 0.946, CI 0.735 to 1.218, *p* = 0.668) in ARB vs non-ARB. Subgroups *H* and *T* refers to reference population; *H* is hypertension, *T* for sample population with mixed comorbidities. *I^2* refers to *I*^2^ as a measure of heterogeneity

Additionally, in a pooled analysis of nine studies that reported discrete data for ACEi, there was no association of critical/death outcomes in patients on ACEi as compared with those not on ACEi (OR 1.008, CI 0.822 to 1.235, *p* = 0.941) as shown in Fig. 3. With regard to patients on ARB, similarly, there was no difference (pooled OR 0.946, CI 0.735 to 1.218, *p* = 0.668) in critical/death compared to those non-ARB (Fig. 4), although for both ACEi and ARB, we might have been underpowered to detect a smaller effect.

## Discussion

The role of RAAS blockers in COVID-19 remains to be fully elucidated, and this has led to significant discussions in the medical communities regarding the safety of these drugs. Whilst multiple national societies supported the continuous use of RAAS inhibitors, we have seen many patients unilaterally stopping them due to concerns after reading the initial reports [22–24]. The emerging outbreak means that there is a need for robust clinical data on these antihypertensives in COVID-19 patients [23].

Our meta-analysis, the largest and most detailed undertaken to date, showed a third of hypertensive and a quarter of overall COVID-19 patients were prescribed an ACEi/ARB, likely due to the increasing risk of infection in patients with comorbidities such as cardiovascular diseases, hypertension and diabetes [8]. Although cardiovascular diseases in combination with COVID-19 portend increased risk of severity and mortality [8, 12], the use of ACEi/ARB is not the likely culprit. The use of ACEi/ARB did not show any association with severity of disease or even death among patients admitted with COVID-19.

On the contrary, this meta-analysis showed that death/critical events may even decrease with the use of ACEi/ARB across pathologies, although the analysis failed statistical significance (*p* = 0.071). This effect however was magnified and was significant among the hypertensive cohorts. Hypertensive patients with COVID-19 who were on ACEi/ARB were 0.67 times less likely to have a fatal/critical outcome than those not on ACEi/ARB (*p* = 0.01). ACEi/ARB was also associated with a significantly lower risk of death (*p* = 0.03) in hypertensive patients. Our results are comparable to another meta-analysis comprising of nine studies and 3936 hypertensive patients. This study demonstrated a lower mortality association of ACEi/ARB treatment in hypertensive COVID-19 patients compared to non-ACEi/ARB (OR 0.57, 95% CI 0.38–0.84, *p* 0.004) [25••]. The benefits of RAAS inhibitors were comparable in both ACEi and ARB. Whilst we did not see a significantly lower risk of death/critical outcomes in patients taking ACE vs non-ACEi and in ARB vs non-ARB, as only a few studies included these data, our analysis might have been underpowered.

Nevertheless, our study in addition to reassuring patients taking RAAS inhibitors begs an important question on whether ACEi/ARB therapy has an obscure beneficial role in patients admitted with COVID-19. Animal studies previously have shown a downregulated expression of ACE2 following SARS infection which results in increased activation of RAAS [13, 26]. This leads to a sequelae of events [13], notably acute lung injury and consequently, adult respiratory distress syndrome (ARDS) [27]. Thus, the use of ACEi/ARB and deactivation of RAAS might be beneficial in preventing this sequence of events [13].

In addition to the benefits of ACEi/ARB in cardiovascular patients [28, 29], our study clearly demonstrates the beneficial effects of ACEi/ARB especially in hypertensive cohort with COVID-19. Whilst the meta-analysis does not modify the existing clinical practice, it provides essential information on the use of RAAS blockers in COVID-19 patients and supports the recommendations of the national medical societies to continue treatment with these drugs [22–24]. Withholding ACEi/ARB could lead to compromising cardiopulmonary reserve in patients who are already at increased risk of COVID-19 [30, 31] which is an important issue for future research and warrants a clinical trial.

### Limitations

Due to the emerging infection, there is insufficient data to compare these analyses to a control population. In order to undertake a comprehensive evaluation of all data on the usage of ACEi/ARB in COVID-19, the search strategy was inclusive. Pre-print data were included which could potentially introduce bias, but at this time of increasing COVID-19 disease, it was pertinent to review all relevant and essential data.

Furthermore, heterogeneity in the meta-analysis is likely due to the varied sample population or different definitions for severity of the disease. For instance, some studies only analysed hypertensive or cardiovascular patients or those of at least ‘moderate’ severity, whilst some are based on hospital inpatients which is likely to be of at least moderate in disease severity. Several steps were taken to decrease heterogeneity; a standard definition of ‘critical’, published by CDCC [19] was used and subgroup analysis of hypertensive patients was done. Additionally, those studies including clinically suspected/confirmed COVID-19 were excluded to keep a comparable group of patients.

### Future Directions

Although our study sheds light on the association between RAAS blockers and mortality in COVID-19, it begs another question as to whether ACEi/ARB lowers the mortality in these patients. There are no clinical data currently on the effect of ACEi/ARB in COVID-19. In order to establish a viable association, future randomised controlled studies are required.

## Conclusion

In conclusion, whilst our meta-analysis demonstrated no association between the use of ACEi/ARB and the severity and mortality among patients admitted with COVID-19, it found evidence of beneficial effects in the hypertensive cohort. As such, we would strongly recommend patients to continue with RAAS inhibitor pharmacotherapy during the COVID-19 pandemic. Further randomised clinical trials are warranted to confirm these findings.

## Electronic Supplementary Material

ESM 1 (DOCX 889 kb)
