# Supplementary material for: Effect of Renin-Angiotensin-Aldosterone System Inhibitors in Patients with COVID-19: a Systematic Review and Meta-analysis of 28,872 Patients
Source: Curr Atheroscler Rep. 2020 Aug 24;22(10):61. doi: 10.1007/s11883-020-00880-6 (PMC7443394; doi:10.1007/s11883-020-00880-6)
Supplement: Supplementary file 1 — (DOCX 889 kb) [file 11883_2020_880_MOESM1_ESM.docx]

Figure 1: Search strategy in Medline


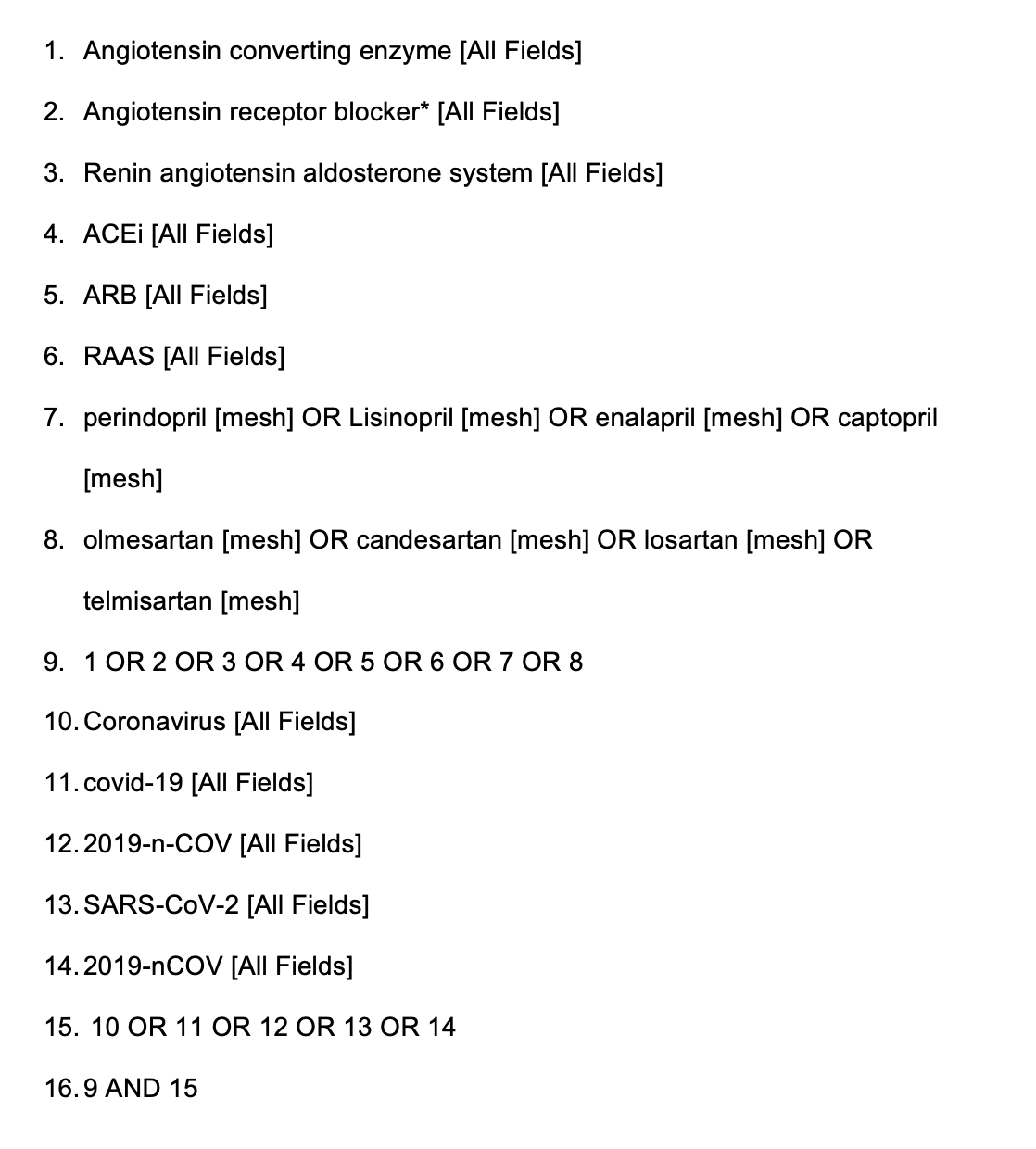


Figure 2: Preferred Reporting Items for Systematic Reviews and Meta-Analyses (PRISMA) flow diagram of the trial selection process


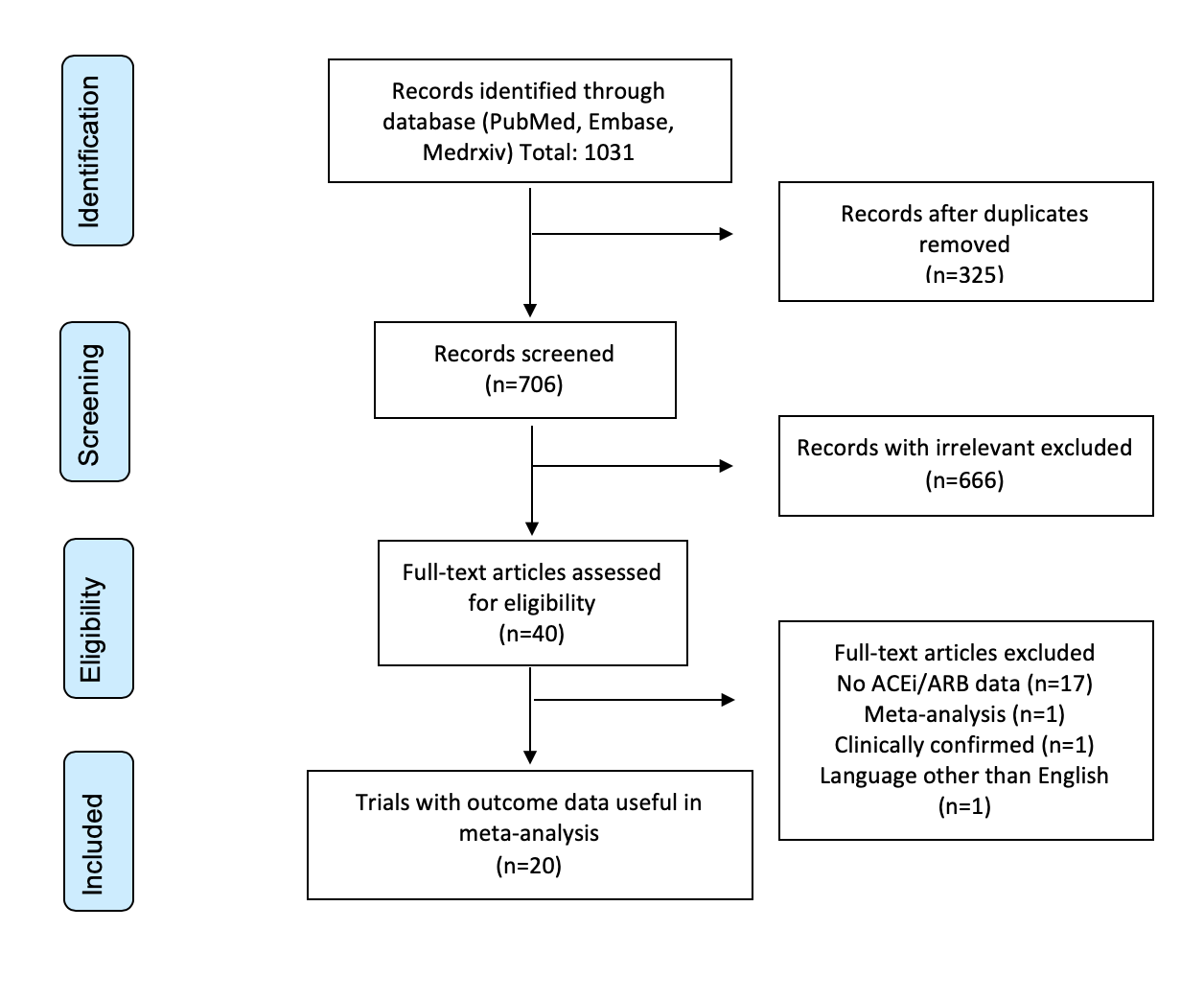


**Table 1:** Newcastle-Ottawa Scale scores for the included trials.

| Authors | Selection | Comparability | Outcome/Exposures | Total | Quality of study |
| --- | --- | --- | --- | --- | --- |
| Abajo | **** | ** | ** | 8 | High |
| Andrea | *** | * | ** | 6 | Moderate |
| Ip | *** | * | * | 5 | Moderate |
| Bean | **** | * | ** | 7 | High |
| Chen | **** | ** | ** | 8 | High |
| Chocdik | **** | ** | ** | 8 | High |
| Dauchet | *** | * | * | 5 | Moderate |
| Feng | **** | * | ** | 7 | High |
| Guo | *** | * | ** | 6 | Moderate |
| Huang | *** | ** | ** | 7 | High |
| Li | *** | * | ** | 6 | Moderate |
| Mancia | **** | ** | ** | 8 | High |
| Mehta | **** | * | ** | 8 | High |
| Meng | **** | ** | ** | 8 | High |
| Reynolds | **** | ** | ** | 8 | High |
| Richardson | *** | * | ** | 6 | Moderate |
| Yang | **** | ** | ** | 8 | High |
| Zhang | *** | ** | ** | 7 | High |

Each star indicates a point for each component. Any study can obtain a maximum of four, two and three stars for each component respectively. Trials with a total score of 7 or higher are considered to be high-quality studies, and those lower than 5 are considered as low-quality studies. These scores indicate at least moderate quality of the included trials.

**Figure 3:** Funnel plot for death/critical outcomes for covid-19 patients on ACEi/ARB


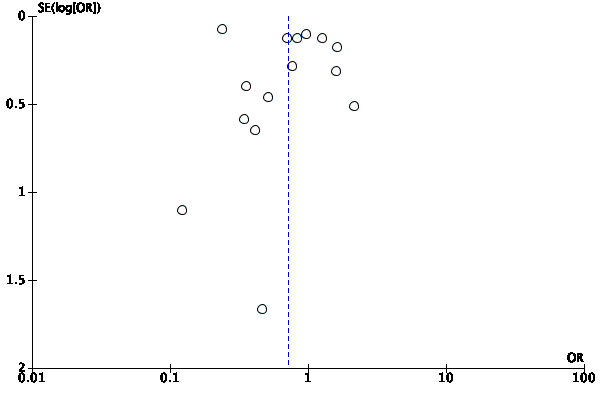


**Figure 3:** Funnel plot for death/critical outcomes for covid-19 patients on ACEi/ARB demonstrating mild asymmetrical.

Sensitivity Analysis (Figures 4-7)

Figure 4: Sensitivity analysis of death/critical events ACEi/ARB vs non-ACEi/ARB (excluding Richardson).


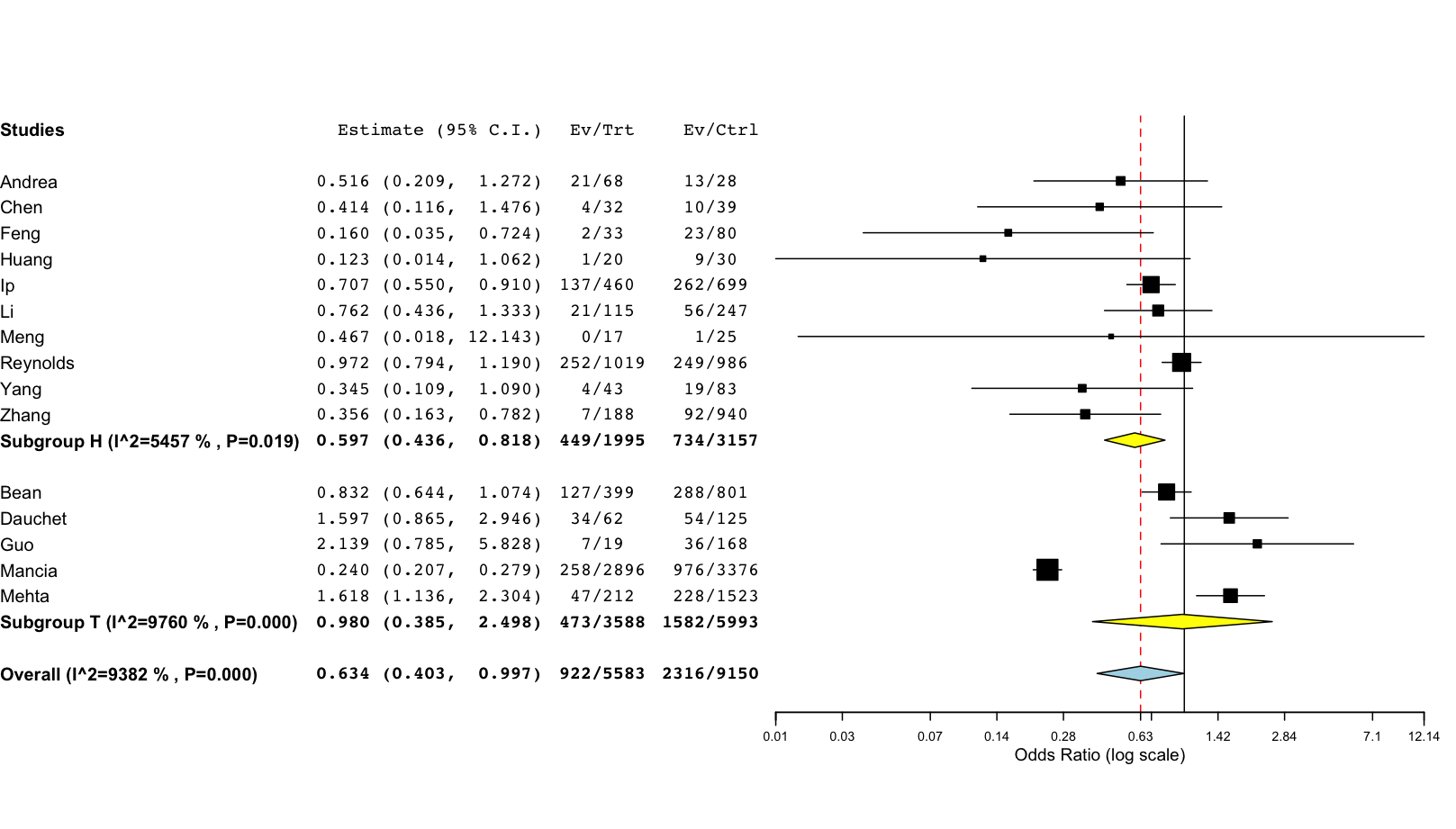


Figure 4: Sensitivity analysis of death/critical events ACEi/ARB vs non-ACEi/ARB patients (OR 0.634,CI 0.403 to 0.997, p= 0.049) in fifteen studies (excluding Richardson).

Subgroup H and T refers to reference population; H is Hypertension, T for sample population with mixed co-morbidities. I^2 refers to I^2^ as a measure of heterogeneity.

Figure 5: Sensitivity analysis of death/critical events ACEi/ARB vs non-ACEi/ARB (excluding Guo).


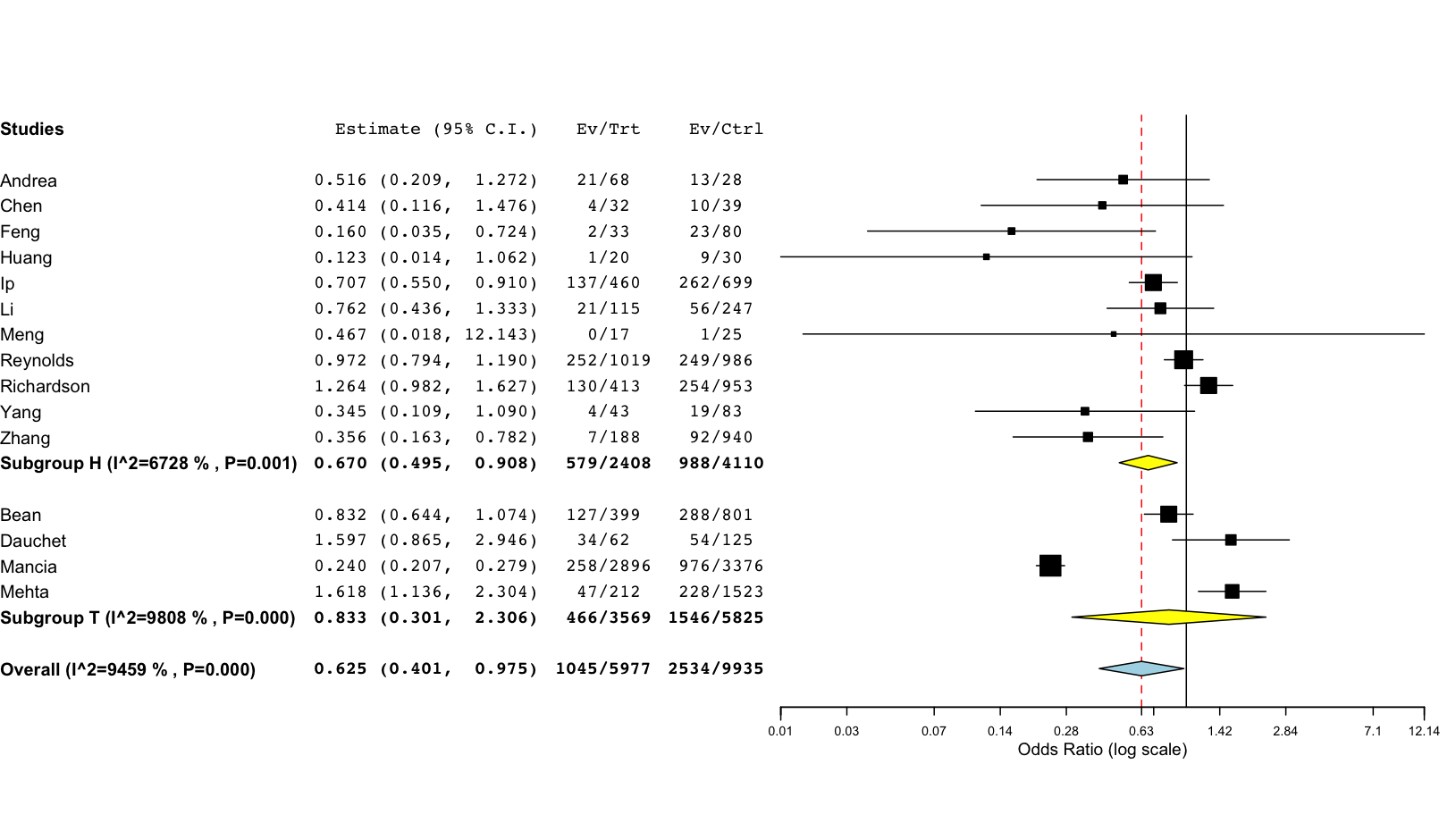


Figure 5: Sensitivity analysis of death/critical events ACEi/ARB vs non-ACEi/ARB patients (OR 0.625, CI 0.401 to 0.975, p=0.038) in fifteen studies (excluding Guo).

Subgroup H and T refers to reference population; H is Hypertension, T for sample population with mixed co-morbidities. I^2 refers to I^2^ as a measure of heterogeneity.

Figure 6: Sensitivity analysis of death/critical events ACEi/ARB vs non-ACEi/ARB (excluding Mehta).


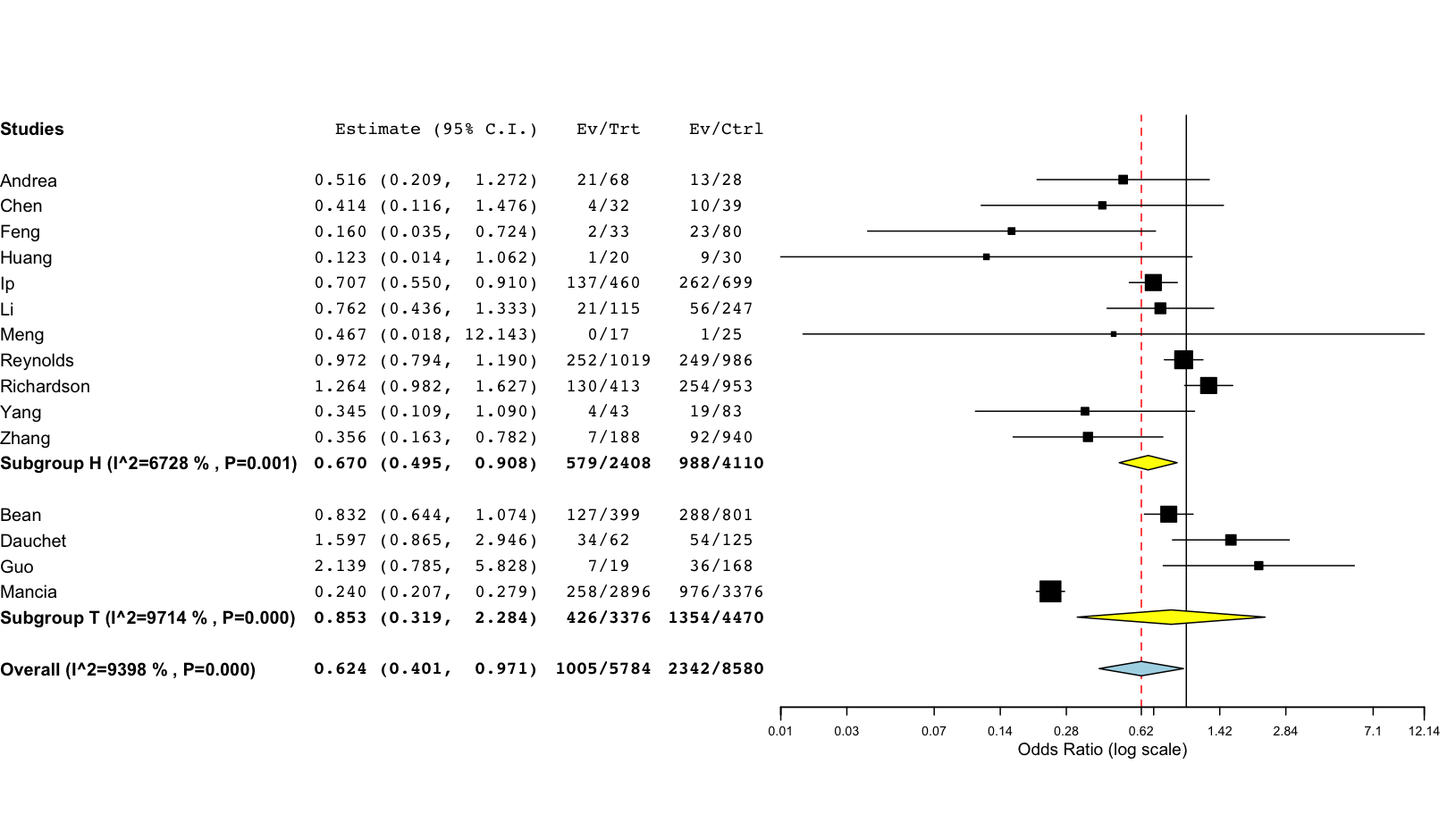


Figure 6: Sensitivity analysis of death/critical events ACEi/ARB vs non-ACEi/ARB patients (OR 0.624, CI 0.401 to 0.971, p= 0.037) in fifteen studies (excluding Mehta).

Subgroup H and T refers to reference population; H is Hypertension, T for sample population with mixed co-morbidities. I^2 refers to I^2^ as a measure of heterogeneity.

Figure 7: Sensitivity analysis of death/critical events ACEi/ARB vs non-ACEi/ARB (excluding Dauchet).


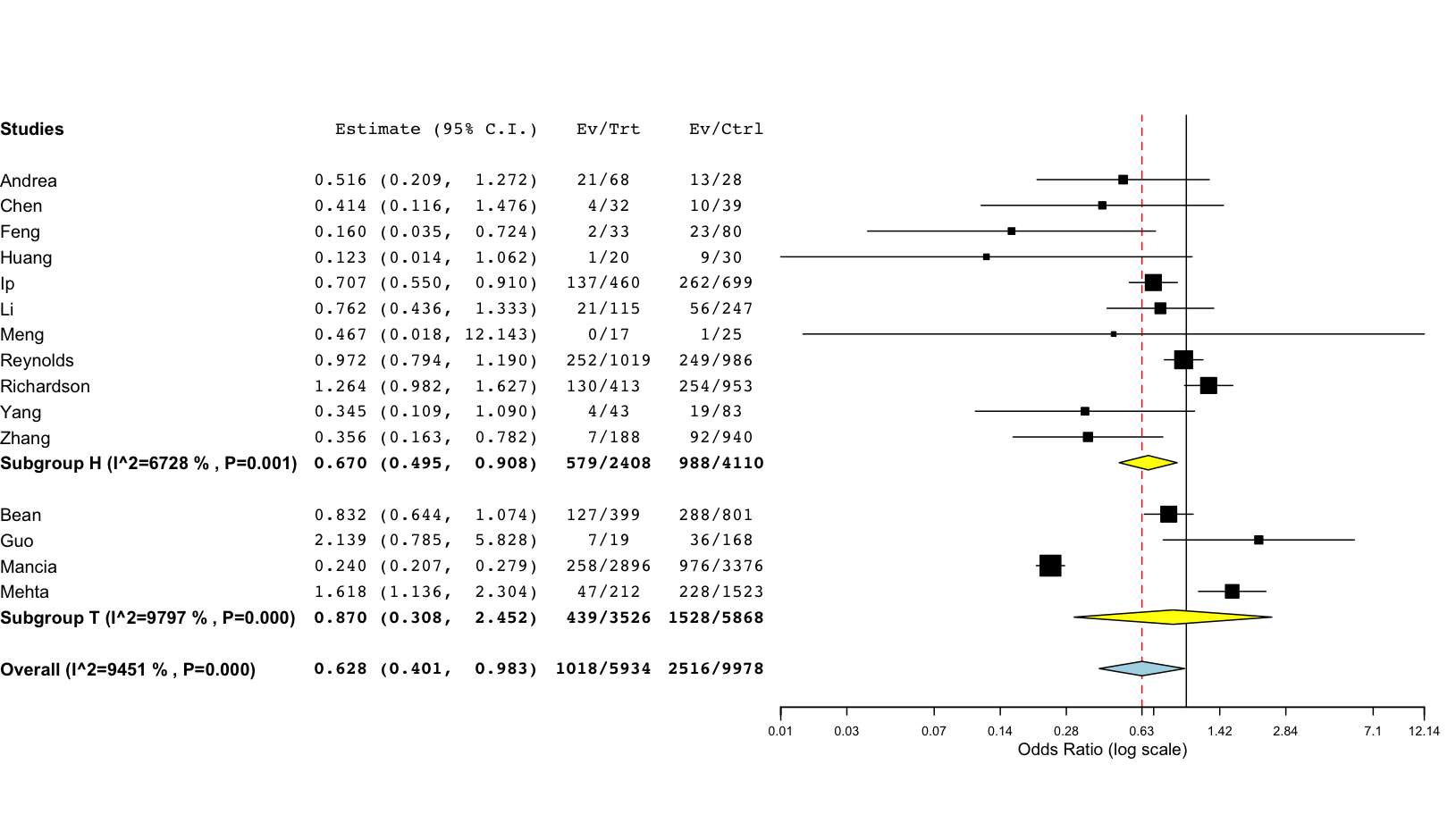


Figure 7: Sensitivity analysis of death/critical events ACEi/ARB vs non-ACEi/ARB patients (OR 0.628, CI 0.401 to 0.983, p= 0.042) in fifteen studies (excluding Dauchet).

Subgroup H and T refers to reference population; H is Hypertension, T for sample population with mixed co-morbidities. I^2 refers to I^2^ as a measure of heterogeneity.
